# Breast Augmentation in Body Contouring Using Autologous Stem Cell-Enriched Fat Grafting: Fifteen-Year Clinical Experience

**DOI:** 10.3390/jcm14165607

**Published:** 2025-08-08

**Authors:** Robert J. Troell

**Affiliations:** Private Practice, Department of Surgery, Touro University Nevada, Las Vegas, NV 89148, USA; rjtroell@gmail.com

**Keywords:** breast augmentation, mastopexy, breast fat grafting, stem cell enrichment, centrifugation–filtration, body contouring, VASER liposuction

## Abstract

**Background**: Variability and low volume yield in breast aesthetic outcomes utilizing fat grafting promoted a search for surgical technique improvement. **Aim**: Using evidence-based information to optimize a surgical technique for aesthetic breast augmentation using stem cell-enriched fat grafting. **Methods**: Retrospective study of consecutive women (n = 118) from 2008 to 2025 requesting breast fat grafting using centrifugation–filtration fat processing combined with platelet-rich plasma and autologous adipose-derived stem cell-enriched fat. **Results**: Most surgical indications were for primary breast augmentation (65.8%), followed by fat grafting after implant removal (13.6%), during or after mammoplasty (13.6%), or simultaneously with implant exchange (12.7%). The mean volume per breast of purified, enriched fat grafted was 192 to 206 cc. Each patient had fat grafted into the subcutaneous plane with some patients having additional fat placed submuscularly in those without a dual plane or submuscularly placed implant, or where an implant capsule was absent. Most patients were either very satisfied or satisfied (95.8%), with 4.2% dissatisfied. Those dissatisfied were mainly those with insufficient breast volume and one with a suspected atypical mycobacteria infection. There was a 11.9% complication rate, with seroma formation at the harvested site the most common at 5.1% (n = 6). Palpable fibrotic areas were second in frequency at 3.4% (n = 4), but with no instances of breast oil cyst formation. The average number of fat grafting sessions per indication was only one, with 6.8% requesting a second staged fat grafting procedure. The revision procedures were only in patients with a sole augmentation indication, except for one mastopexy patient with severe breast size asymmetry. An estimated 75–85% grafted volume take was confirmed by a previous diagnostic ultrasound measurement study. **Conclusions**: Breast fat grafting incorporating learned knowledge of optimal harvesting, processing, storing, enrichment, and administration techniques yielded superior consistent breast enhancement aesthetic outcomes with a high patient and surgeon satisfaction rate through increased adipocyte survival, while minimizing complications including a low incidence of fibrotic areas and no oil cyst formation.

## 1. Introduction

Liposuction is the most requested cosmetic body procedure in the United States, followed by breast implant augmentation [1]. Improvement of breast size and shape in primary breast augmentation or in breast reconstruction after implant removal, after mastopexy, after reductive mammoplasty, or associated with breast cancer therapy are mainstays of current cosmetic and plastic surgery procedures. Because of the concern with synthetic alloplastic implant risks and the high complication rate (10–25%) and high revision rate [2,3,4,5], alternative treatment has been sought. Breast fat grafting as the primary goal in breast augmentation or as a breast reconstructive procedure has gained popularity due to the advantages of its autologous nature, being readily available and low risk, its low revision rate, and because it can be completed without implementing general anesthesia, but with the use of oral sedation and a wetting solution (lidocaine, epinephrine, and bicarbonate) safely and comfortably [6].

In the past, widespread use of breast fat transfer was delayed by the initial concerns of cancer surveillance issues and inconsistent aesthetic outcomes [6]. Ubiquitous use of natural breast augmentation using fat was delayed because of the concern of altering a radiologist’s ability to interpret a mammogram during cancer surveillance. Microcalcification formation from adipocyte death may increase false positive diagnosis [7,8,9]. Rubin et al. in a blinded radiologist study revealed that the presence of macrocalcifications from breast fat grafting was six times less than that observed in interpretation post-reduction mammoplasty [10]. Breast data system and imaging reporting scores were less favorable with reduction mammoplasty [10]. In summary, fat grafting to the breast does not adversely affect radiologist mammogram cancer surveillance.

Scientific advances continue to yield improved fat viability, translating to optimal aesthetic results in patients requesting natural breast augmentation, while minimizing side effects and complications. Evidence-based medicine continues to evaluate technologies and techniques that optimize fat harvesting, processing, enrichment, storage, and administration (injection) with the goal of the most consistent fat grafting outcomes with superior physician and patient satisfaction rates [6]. Fat grafting has the most varied inter-surgeon technique than any other cosmetic surgical procedure of the face or body.

## 2. Methods

### 2.1. Patient Selection and Decision-Making Process

All patients underwent a detailed initial aesthetic consultation, medical clearance, and jointly arrived at a surgical plan. A mammogram is attained and required preoperatively.

**Figure 1:**
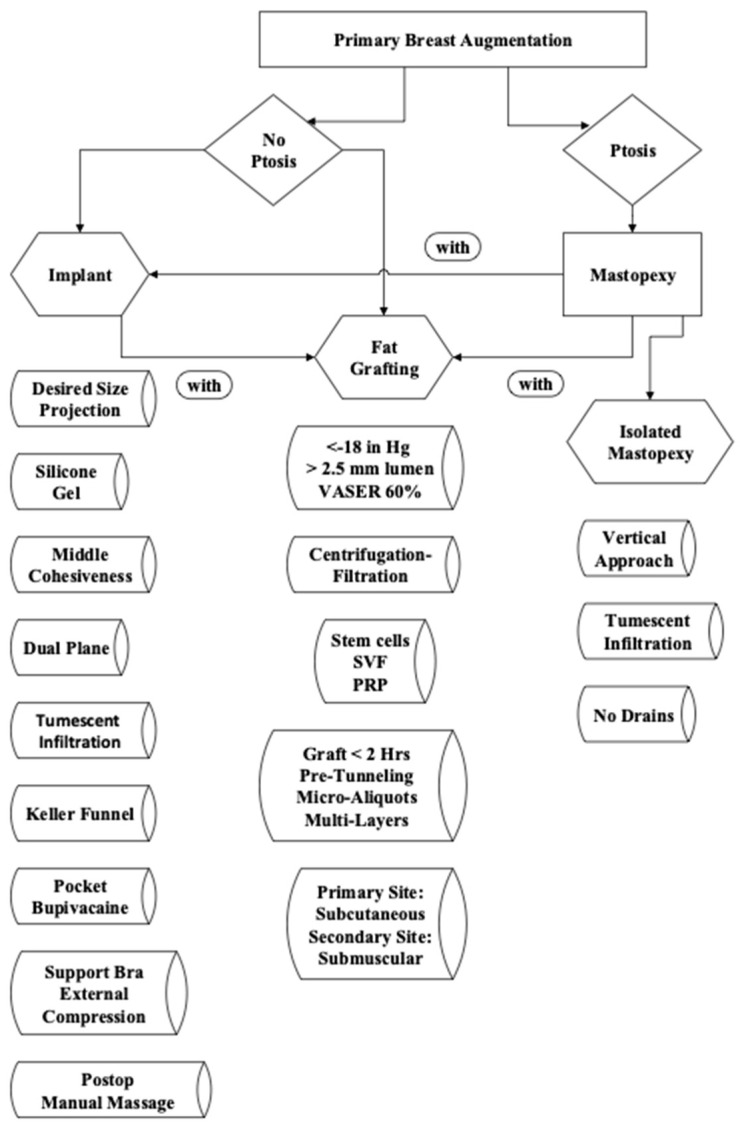
Primary breast augmentation flow sheet.

A primary breast augmentation proposed flowchart differentiates a diagnosis of breast ptosis with the lack of ptosis [11]. Vertical mastopexy was the preferred treatment technique over Wise pattern (inverted-T) for treating ptosis, performed simultaneously in mostly silicone gel implant patients or with a staged approach in breast fat grafting. Breast augmentation is accomplished by silicone gel implant placement, breast fat grafting, or less commonly, composite augmentation (implant and fat transfer simultaneously or staged) with or without mastopexy (Figure 1) [11].

Proposed breast augmentation algorithm to assist in forming a breast aesthetic surgical plan with an option of fat grafting [11].

Clinical indications for autologous breast fat grafting are numerous and are always performed simultaneously with advanced body contouring or liposuction (Table 1) [6]. All patients underwent a discussion highlighting that the most significant limitation of fat grafting was an outcome of no more than one half to one breast size. Also, they were informed that about 6–8% of women prefer a second-staged fat grafting procedure for additional breast volume [6].

The American Society of Plastic Surgery (ASPS) has given its guidance from 1987 through 2012 with policy statements regarding the topic of breast fat grafting. Although it took 25 years, the policy statement in 2012 noted: “autologous fat grafting is an effective option in breast reconstruction following mastectomy while demonstrating moderate to significant aesthetic improvement” [6,12]. Additionally, the Food & Drug Administration (FDA) has published guidelines on the use of human cells and tissues. These updated 2020 guidelines are related to the homologous use of these autologous substances and defining minimally manipulated tissue processing. The FDA allows the use of autologous stem cell-enriched adipose tissue for fat grafting to the breast [13].

Patient selection is always paramount in any surgical procedure for achieving optimal outcomes with minimal complications. Certain medical issues may lower adipocyte cellular viability with fat transfer (Table 2). Candidates for breast fat grafting when individuals display an underlying medical condition that lowers the fat survival rate was assessed depending on the severity, duration, and combination of these variables. The FDA banned the sale of textured coated breast implants (2019) because of an increased risk of the implant capsule promoting the development of anaplastic large cell lymphoma (BIA-ALCL) [6].

This is a retrospective review of a 15-year experience evaluating consecutive women opting for breast fat grafting for any of the clinical indications. All patients undergoing body contouring or any face or body fat grafting procedure were recommended to be at their ideal body weight. Standard weight loss strategies were presented at the initial consultation and it was recommended that they be attempted prior to body contouring. The strategies included dietary changes, muscle strengthening and aerobic exercise, bioidentical hormone supplementation, and more recently, the addition of GLP-1 agonists versus bariatric weight loss surgery. Weight loss both lowers surgical risk and increases the likelihood of achieving a better cosmetic outcome. All patients were ASA class I or II and during a comprehensive medical preoperative work-up determined safe for surgical therapy. All patients underwent a detailed informed consultation and agreed to proceed with the breast fat grafting and body contouring procedures.

### 2.2. Harvesting

The goal of each step in fat grafting to the breast is to minimize adipocyte trauma. Harvesting techniques that protect adipocytes and protect against stem cell injury are the first steps to a successful outcome (Table 3) [6,14]. Wetting solutions containing lidocaine with epinephrine did not negatively affect adipocyte survival, although there is a suggestion that lactated Ringer’s (LR) is preferred over normal saline (NS) for adipocyte survival [15,16]. There are no studies in the literature showing improved fat cell survival related to any specific anatomical donor site [17]. However, this clinical experience notes a significantly less amount of fibrous tissue and bleeding from the inner thigh donor site, which makes the fat purification process easier than other anatomical areas. However, there is caution in the inner thigh area, where there is a higher risk of indentations and more skin laxity resulting after fat harvesting than in other body areas.

Harvesting with 4 mm diameter cannulas revealed a greater number of viable adipocytes compared to 2 or 3 mm cannulas [18]. Vented cannulas, known as VentX (Bausch Health, Laval, QC, Canada), have a small hole at the cannula base, producing a continuous leak in the cannula. Studying the biophysics of the vented cannula reveals a reduction in both fluid viscosity and suction forces injuring adipocyte cell membranes. Lower pressure suction creates fewer sheering forces that lower cellular injury. Studies have concluded that suction about two-thirds of maximum at sea level (−18 in Hg) [19] and liposuction cannulas larger than 2.5 mm reduce cellular trauma [18,20].

In this study, all the harvesting recommendations in Table 3 were implemented to include 3.0, 3.7, and 4.6 mm vented liposuction cannulas and suction less than −18 in Hg. In most patients, an autoclavable glass Origins Fat Collection Container (Bausch Health, Irvine, CA, USA) was used. Superwet infiltration was preferred over a tumescent technique in order to treat more anatomic areas without reaching toxic lidocaine doses. Maximum lidocaine dosing was calculated at 50 mg/kg in patients (55 mg/kg in morbidly obese) augmented by oral sedation and analgesia with some requiring intravenous conscious sedation. Less postoperative bruising and less intraoperative blood loss was observed using an epinephrine wetting solution concentration of 1.5 mg/L (initially using a standard Klein solution of 1 mg or 1 cc of 1:1000 epinephrine per liter) [21].

### 2.3. Ultrasound-Assisted (VASER) Energy Delivery

Body contouring is performed simultaneously with breast fat grafting. However, the technique of high-definition ultrasound-assisted liposuction must be changed to prevent a suboptimal donor fat graft [6,22]. Instead of delivering maximum ultrasound energy for fat removal and skin tightening, the amount of energy and duration of ultrasound delivery requires alteration. Lowering the amount of ultrasound energy did not significantly affect body contouring positive effects (more skin retraction and less blood loss), but does lower stem cell and adipocyte death.

Schafer et al., using VASER third-generation ultrasound at 60% energy and pulsed mode, revealed high cellular viability of adipose-derived stem cells (ASCs) and adipocytes—87% and 85%, respectively (Bausch Health, Laval, QC, Canada) [23]. The lipoaspirate samples were processed immediately after aspiration by a good manufacturing practices (GMP) laboratory (Cytori Therapeutics, Inc. San Diego, CA, USA). A lipolysis assay was chosen to evaluate cellular (adipocyte and stem cells) viability and to determine if they were functionally active [23].

Another clinical study using the same energy parameters and liposuction technique by Troell showed even higher survival rates of both stem and fat cells—97% and 92%, respectively [14]. The adipocytes and stem cells were assayed by two independent GMP laboratories, American Cryostem and Touro University Nevada.

Fischer et al. [24] showed histologically, with the addition of filtration (500- and 800-micron filters), that 80% of human fat transplanted into mice survived. A Stanford University clinical fat harvesting study concluded that the stem cells harvested using ultrasound kept their pluripotency [25]. Ultrasound’s “acoustic streaming” creates smaller fat aggregates, which allows better neovascularization of the smaller grafted fat aliquots.

Summarizing, VASER lowers blood loss from between 26 percent less [26] to up to six times less [27] compared to suction-assisted liposuction (SAL). The process provided 53% more skin tightening compared to suction-assisted liposuction, while that [26] allowed for high-definition sculpting [6,22] and a high cellular survival rate of harvested fat and stem cells [14,23,24]. In this study, all patients having breast fat grafting underwent VASER high-definition at the recommended energy levels stated using a 5-ring solid ultrasound probe.

### 2.4. Processing

Once the fat is harvested, processing, enrichment, and administration techniques result in a superior final breast volume (Table 4) [6,14]. From the author’s survey, more than 90% of cosmetic and plastic surgeons do not perform any processing or enrichment of grafted fat. Processing best practices purify fat by alleviating oil, dead adipocytes, and red blood cells while compacting harvested fat by excess wetting solution extraction.

The clinical experience initially implemented the LipoKit, and later the 416-centrifuge (MediKan, Co., Ltd., Seoul, Republic of Korea) along with the manufacturers patented 100 μ filter and weighted TP-101 60 cc syringes for fat processing for all aesthetic face and body fat grafting procedures [14].

Evidence-based clinical studies have provided the basic knowledge to implement this purification technology. Kurita et al. determined the optimal centrifuge settings (3000 rpms for 3 min) for fat purification and excess centrifugation settings resulted in less adipocyte loss with separation of ASCs and stromal vascular fraction (SVF) cells [28]. Troell implemented these centrifugation setting recommendations and scientifically confirmed the superior number and viability of stem cells and fat cells during breast fat transfer [14].

Some surgeons prefer to wash harvested fat. However, despite the washing removing some blood, oil, and cellular debris, this fluid both dilutes the concentrated fat and washes away adipose-derived growth factors. Cytori Therapeutics (San Diego, CA, USA) studied the efficacy of a washing-filtration system they developed known as PureGraft^®^. The system achieved a reduction of oil by 1–5% and wetting solution reduction by about 5% without a reduction in fat cell numbers (and personal communication 2014) [14]. The surgeon who reinvigorated face and body fat grafting, Sydney Coleman, MD, cautioned that the mechanical disruption as a result of fat washing negatively alters the fragile fatty tissue architecture [29,30].

Troell compared a centrifugate-filtration device (Lipokit) to a double filtration-washing system (PureGraft). Breast volume using centrifugate-filtration resulted in 40% more thickness initially (at 10 weeks) with 29% more thickness long-term, measured by the Terason10 MegaHz diagnostic ultrasound device [14]. The ultrasound 3D measurement of final breast fat volume revealed 75–85% end fat grafted volume enhancement [14]. This objective measurement correlated to the patient and surgeon subjective visual assessment of volume change [14].

**Figure 2:**
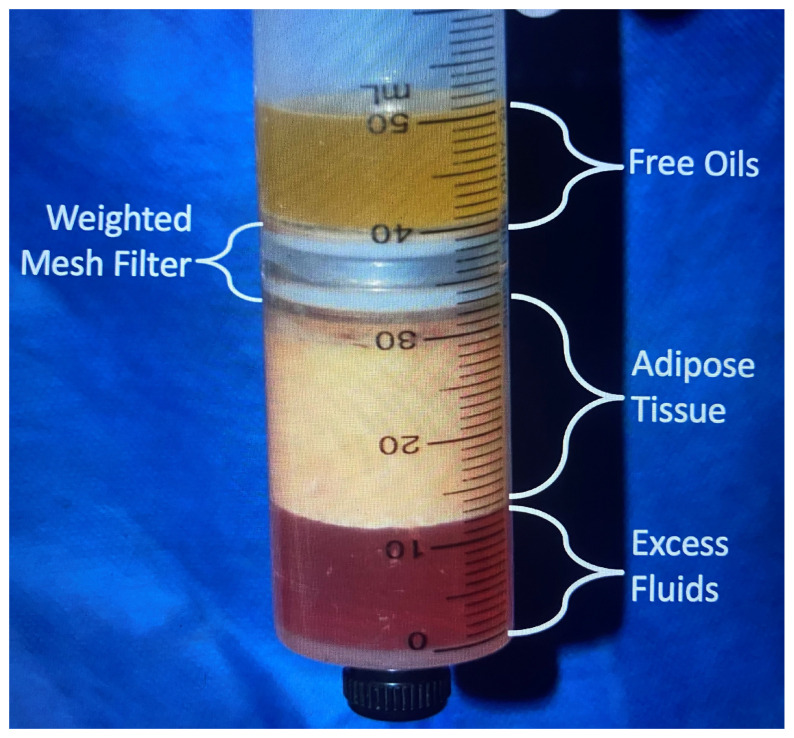
Centrifugation–filtration TP 101 syringe from Medikan, Co. (Seoul, Republic of Korea) A 100 μ filter inserted into the weighted 60 cc syringe allows purification of harvested fat. Centrifugation at 3000 rpms for 3 min forces oil through the filter into the top of the syringe. The inferior pellet of the infranatant (1 cc) contains growth factors, stem cells, and stromal vascular fraction cells. The infranatant is blood and wetting solution squeezed out of the compacted fat. The pellet is then added to the purified fat along with 1 cc of platelet-rich plasma (PRP) prior to administration. (Image from a surgery using a MediKan, Co. TP-101 patented syringe after centrifugation).

In this breast fat grafting patient population, all patients had centrifugation–filtration on all grafted fat implementing the MediKan system. Gravity separated fat from the harvesting container was transferred to a Toomey syringe and using an adapter injected into a Luer lock 60 cc syringe. This harvested fat was then transferred with an adapter into four patented, weighted 60 cc syringes containing a 100-micron filter and placed into a large centrifuge (LipoKit or 416D) programmed at 3000 rpms for 3 min. These settings squeeze out wetting solution to compact the unprocessed fat into 30 cc (25 to 35 cc) of fat volume. Additionally, the most dependent infranatant aliquot (1 cc pellet) contains approximately 4 million SVF cells and 1 million ASCs, measured by two independent good manufacturing practices (GMP) laboratories (Figure 2) [14,31].

### 2.5. Enrichment

#### 2.5.1. Background

The decision to incorporate enrichment into fat grafting begins with understanding the complex extra-cellular biological processes and cellular influences in the human body. The basics of cellular survival is sufficient oxygen and glucose delivery to the cell. Removing an adipocyte from its donor site and transferring it to a recipient bed cuts the supply from blood vessels. An adipocyte is thought to survive for 24 to 48 h without a blood supply, while stem cells may survive up 3 to 5 days [31]. Injecting a fat graft into the extracellular matrix initiates neovascularization along the matrix scaffold stimulated by a complex plethora of cellular interactions and cytokine effects. The labyrinth of cellular processes interacts to affect cellular viability [6,31].

#### 2.5.2. Platelet-Rich Plasma (PRP)

Platelet-rich plasma contains 30 blood-derived growth factors with the most impactful one in fat transfer being vascular endothelial growth factor (VEGF). This growth factor stimulates blood vessel formation in the first 48 h post-grafting. Studies have shown that injecting PRP into the skin and subcutaneous layer assists in increasing the following: adipocyte survival, ASC numbers (four-fold), extracellular fibrin matrix (ECM) formation, and micro-capillary networks [32].

#### 2.5.3. Autologous Adipose-Derived Stem Cells

##### Discovery

Zuk [33] in 2001 was the first to show that stem cells mainly reside in in the fat layers of one’s body, in a concentration 2500 times higher than in bone marrow. Subsequently, others have successfully used these ASCs and SVF to improve fat grafting aesthetic outcomes [6,14,23,24,31].

##### Method of Action

The complex interaction of cells, growth factors (GF), and cytokines have been elucidated in the cellular milieu in the recipient bed related to fat transfer [6,31]. Resident and grafted stem cells along with WBCs are chemotactically attracted to the hypoxic, inflammatory area. Platelet-derived growth factors (PDGF) among other cytokines stimulate ECM formation binding fibrin monomers [31,32]. Blood vessels grow along this fine scaffold architecture to the grafted adipocyte. Additionally, transforming growth factors (TGF) derived from platelets induce cellular proliferation and DNA synthesis [34]. Resident fibroblast GFs, known as heparin-binding GFs, result in neo-angiogenesis and grafted cellular survival [31,32].

One fat transfer theory stated that most of the adipocytes grafted survive. A contrary theory, suggested by fluoresceine tagging stem cell membranes, concludes that most grafted adipocytes die [31]. Then, stem and progenitor cells transform into an adipocyte [31].

##### Clinical Studies Verifying Stem Cell Benefits

The first study to evaluate if ASCs may be of benefit in breast surgery opened the way to other clinicians. Patients (n = 21) underwent partial mastectomy and radiation therapy (XRT) [35]. Breast reconstruction used stem cell-enriched fat grafting. The study found a high patient satisfaction rate, minimal complications, and that ASC fat grafting yielded adequate breast tissue thickness [35].

Subsequently, there was a multi-center European Union clinical trial treating breast defects from T_2_NoMo breast cancer patients (n = 71) surgically treated +/− radiation therapy, called RESTORE 2 [36]. Syringe collection fat harvesting used an automative ASC processing system (Celution^®^ system, Cytori Therapeutics; San Diego, CA, USA). MRI anatomical assessment (blinded radiologists) revealed an 85% positive outcome. Complications included breast cysts (14.9%) only. This confirmed the procedure safety with no evidence of inducing cancer recurrence using ASCs in breast fat grafting [36].

Stem cells are more robust than adipocytes. They have the ability to transform into vascular mural cells or endothelial cells, or to stay as ASCs [31]. Stem cells can be harvested, processed, and concentrated from the lipoaspirate as with the use of the MediKan system employed by the author [4,14,31,37]. When ASCs (7–21 μ) differentiate into adipocytes (70–140 μ), the younger adipocytes are more robust than mature cells, resisting cellular trauma better. Smaller cellular diameter allows for placement via smaller sized cannulas [31].

##### Stem Cell Policy Statement

The ASPS has presented a policy statement on stem cells in cosmetic surgical procedures [38]. The policy statement was based on a systematic review of the peer-reviewed literature. The joint task force concluded: “while there is tremendous potential for the future use of stem cells in aesthetic surgical procedures, the scientific evidence and other data are very limited in terms of assessing the safety or efficacy of stem cell therapies in aesthetic medicine” [38]. Autologous stems cells attained through the harvesting and processing of fat are allowed by the FDA for fat grafting enrichment [13]. Yoshimura coined the term cell-assisted lipotransfer (CAL) when adding ASCs [33,39].

In this study, all patients underwent breast fat transfer enriched with PRP, stem cells, and SVF cells. In general, the first one cc of infranatant after centrifugation and one cc of PRP was added to each 25 cc of purified centrifuged fat. This methodology mirrored that discovered and upgraded by Katori Yoshimura (and personal communication) [39].

### 2.6. Storage

Since revision surgery is contemplated in 7–10% of breast fat grafting patients, the options include storing harvested fat for later administration or acquiring new fat at the time of the revision. Procedures with additional fat grafting may be desired. Storage can be used for fat aliquots or for stem cell expansion for later therapeutic uses [31]. Best practices in stored fat require strict adherence to a regimented storage process to include an added cryo-preservative, disciplined freezing rate, and liquid nitrogen storage at 4 degrees C to maintain cellular viability [40,41,42].

Wolter et al. noted preservation of half of baseline cellular metabolic activity after adding a cryopreservative substance, in contrast to losing 93% of activity with freezing alone [41]. Moscatello et al. [40] revealed minimal adipocyte survival and complete ASC death with instant freezing at −20 °C. Frozen samples with each of six different cryopreservative substances showed cell growth in culture. The authors stated that a disciplined freezing rate adding a cryopreservative maintained cellular survival [40].

Matsumoto et al. [42] studied viability of both ASCs and adipocytes at varying storage temperatures and concluded that fat persisting in a collection container for 4 h resulted in high fat cell death, yet ASC viability was preserved. They also noted that freshly harvested fat fared better than cryopreserved fat. Confirming the results of Wolter, Moscatello et al., and Matsumoto, Son et al. showed the requirement in fat and ASC storage of a cryoprotective substance [43].

Evaluating the effect of cryopreserved fat grafts treated with either normal saline alone, SVF, or ASCs in nude mice, Bae et al. revealed the ASC specimens had significantly increased graft volume and weight with histological proven cellular survival [44]. In summary, ASCs added to cryo-banked fat yielded high (>90%) fat cell viability [6,40,41,42].

In this study, patients were given the opportunity to store any unused harvested fat. Most patient had all the harvested and processed fat used in breast fat grafting and many had buttock and hip fat transfer during the same procedure. All patients who stored fat later used this stored fat for additional fat breast augmentation.

### 2.7. Administration

The timing from initial fat harvesting to recipient bed injection is the initial decision. Matsumoto et al. [45] showed superior fat cell and ASC survival at one hour compared to two hours, while harvesting at 4 h before fat administration dramatically lowered fat cell yield. Compared adipose tissue cell viability from one, two, and four hours post-harvesting. Their conclusion was to perform fat transfer as soon as it was harvested, but with the longest delay of two hours [6,45].

Micro-aliquot injection of no greater than 2.5 to 3 mm in diameter increases the chance of graft revascularization. The addition of multiple layering from the skin down to the muscle fascia or above glandular tissue (i.e., the breast) into the ECM optimizes the ability of cells to receive a blood supply before cell death occurs [29,46,47]. The goal of fat administration is maximum volume without reaching the peak of the curve where more fat results in loss of adipocytes from the inability to revascularize. Avoiding excess volume of fat grafts in a specific site will minimize vascular necrosis from lack of neovascularization using the recommendations of Coleman [29,30], Ersek [46], and Carpenada [47].

In this study, all patients had centrifuged–filtered purified fat enriched with stem cells, SVF, and PRP administered within two hours of harvesting, with most patients undergoing administration within one hour. The injection was completed with a 3.0 mm single hole, Coleman fat harvesting cannula into the *subcutaneous* space down to a plane just above the firmer parenchymal glandular breast tissue. Patients who had additional fat remaining after the subcutaneous placement, and who did not have a present breast implant or implant capsule present, underwent *submuscular fat* transfer through an incision at the inferior-lateral inframammary crease.

The submuscular space is immediately below the pectoralis major muscle and above the chest wall and pectoralis minor muscles. Most of the fat was injected into the superomedial quadrant where the most aesthetic changes would be appreciated. This space has fibroareolar tissue and some muscle attachments between the two muscle plans. This space allows for neovascularization if one avoids excess fat volume (estimated about 150 cc) administration. Intramuscular fat administration was not performed for two reasons: (1) the muscle is fairly thin (2–3 cm), which would allow only a limited volume, and (2) nearly all patients were treated under oral and conscious sedation, and intramuscular injection would have caused excess discomfort. The veins in the pectoralis muscle are small compared to the gluteus maximus, so the risk of fat embolism is negligible.

Anesthesia and hemostasis were achieved by the administration of 75–125 cc of wetting solution infiltrated with a blunt infiltrating cannula into the breast areas to be grafted.

#### 2.7.1. Breast Capsular Contracture Necessitating Implant Removal

Patients who presented with Baker grade III or IV capsular contracture (all implants were in dual plane) were given the following options: (1) total capsulectomy and replacement of an implant without or adding a scaffold (acellular dermal matrix, Alloderm or Strattice, or absorbable monofilament 4-hydroxy-butyrate- GalaFLEX), (2) trial of fat grafting to decrease severity of capsular contracture (no patients elected this option), or (3) total capsulectomy, implant removal, and breast fat grafting [6,11].

When a contracted capsule was excised, it was recommended to stage fat grafting for at least three months for several reasons: (1) to achieve an aesthetic end result with fewer changes occurring from the healing process, (2) to alleviate the acute inflammatory response that can be toxic to grafted fat, (3) to undergo the procedure under a safer conscious sedation anesthesia than general anesthesia, and (4) so that tissues planes are healed with edema resolution to allow optimal neovascularization along the extracellular matrix scaphoid. However, if the degree of surgical dissection resulted in minimal trauma to the surrounding soft tissues, then fat grafting proceeded at the time of implant removal and capsulectomy [6,11].

#### 2.7.2. Breast Asymmetry Patients

Patients who had asymmetric breasts could be treated with fat grafting alone, combined with implant surgery, or with additional skin or soft tissue surgery. The additional procedures were to either produce some skin tightening or breast reduction, most commonly at the lateral chest/axilla or inferolateral breast itself.

Showing the efficacy of fat grafting over implants for asymmetric breasts, a prospective study comparing those treated by breast implants compared to fat grafting in breast asymmetry treatment (n = 35) revealed breast implant surgery averaged 2.1 operations to achieve symmetry with a 26% complication rate and 26% converting to fat grafting [48]. Fat grafting alone achieved symmetry in 86% with no major complications and 9% proceeding to have additional implant surgery. They concluded that breast reconstruction with implants theoretically was a single-stage procedure but clinically resulted in enough revisions to double the number of surgeries required to achieve the aesthetic goal, with a high complication rate. Breast fat grafting offered a durable result with a smaller number of surgeries and minor complications, while achieving long-term volume and symmetry [48]. However, the ultimate desired size or appearance may not be attenable with fat grafting alone, depending on the patient’s body habitus and quantity of fat available.

VASER ultrasound liposuction alone or combined with Renuvion helium-based plasma radiofrequency technology has successfully both reduced overall or subunit breast size and resulted in satisfactory skin tightening.

#### 2.7.3. Breast Implant Removal Patients

When combining breast implant removal simultaneously with fat grafting, Del Vecchio referred to this surgery as “simultaneous implant exchange with fat” (SIEF) [49]. Those patients who elected SIEF typically did not remove the capsule of the implant. The indications for capsulectomy (total or near-total) were (1) abnormally thickened capsule and (2) capsular contracture. When capsulectomy was performed, the fat grafting was performed later, no sooner than 6–8 weeks to allow the potentially cellular damaging inflammatory response to subside. When removing an implant while keeping the capsule, to prevent the “window shading effect” from the capsule edge, the capsular incision edges are reapproximated with a running absorbable suture.

Simultaneous fat grafting with implant placement can provide a single-stage surgery with minimal downtime. Auclair and Anavekar placed subglandular implants in the subfacial plane (n = 190), followed during the same procedure by fat grafting. The site of fat grafting was mainly at the superior and medial aspects of the breast (mean 125 cc) with only 5% requiring an additional fat grafting session. There were no infections or hematoma formation, and only two patients (1%) had capsular contracture [50]. Placement of fat into the breast with an implant present is much safer with a higher degree of fat survivability when the implants are in the submuscular or dual plane and the grafted fat into the subcutaneous area to avoid implant puncture.

In this study, those patients having fat grafting with an implant remaining in place all had submuscular implants (>95% silicone gel) and had the fat injected with blunt 3.0 mm cannulas only in the subglandular (subcutaneous) plane. Most grafted fat was placed in the breasts superior pole, especially in the superior-medial quadrant.

#### 2.7.4. Breast Mastopexy and Reduction Mammoplasty Patients

Those patients who had previously undergone a mastopexy or reduction mammoplasty had fat placed in selected areas to enhance the breast shape and/or improve symmetry or had the entire breast grafted for volume augmentation. Those mammoplasty patients who requested additional volume, were without implants, and had adequate volume of harvested/processed fat underwent fat transfer into both the subcutaneous and submuscular planes. Some of these patients also requested either internal skin retraction using VASER and Renuvion or external skin tightening by using either VASERShape therapeutic ultrasound or microneedling radiofrequency (VenusVivaMD).

### 2.8. High-Definition VASER Liposuction and Renuvion Helium-Based Plasma

High-definition VASER liposuction was begun by Troell in 2011, adding Renuvion helium-based plasma technology (HBT) (Apyx Medical Corp., Clearwater, FL, USA) in 2018 for additional skin tightening. HBT has been shown to increase skin tightening by initially contracting collagen fibers by 65% of the fibroseptal network [51], followed by delayed skin tightening with healing approaching 30% [52,53,54]. These technologies achieved superior body contouring outcomes and were also used for lateral chest and breast skin tightening and as a breast reduction alternative technique. If the patient has volume deficiency in other body sites, most commonly the buttock or hip area, fat grafting and/or body silastic implants using either standard or patient-specific (custom) were discussed.

### 2.9. Patient Follow-Up

Coleman’s long-term patient follow-up experience observed minimal fat absorption and remodeling after two to three months post-transfer [29,30]. He observed stable volume up to 12 years post-grafting. These observations were validated by the longevity data from the cohort of Troell, revealing stability after 3 months from administration, unless patients gained or lost weight [6,55]. Delay et al. confirmed these two observations of volume stability after four months with stable body weight [56].

Patients were instructed to follow-up at 1 day, 1 and 2 weeks, 1, 2, 3, and 6 months, and every year thereafter. Their estimate of final breast volume was first assessed at 3 months. Photographs, patient physical examination, weight measurement, and directly querying were used to assess breast volume. Previously, objective breast volume was measured using 3D diagnostic ultrasound [14].

The Global Aesthetic Improvement Scale Assessment (GAIS) was the patient reported outcome measure implemented in this study This five-point scale (0 to 5) is from worse, no change, improved, much improved, and very much improved. Those who experienced weight gain were counseled on weight loss to include dietary changes, exercise, and bioidentical hormone testing and supplementation. Patients were recommended not to return to exercise for at least 3–4 weeks after surgery due to the increased risk of seroma formation from the liposuction.

Those requesting revision surgery or additional breast augmentation were presented with the following options: (1) repeat breast fat grafting (if sufficient body fat remained or for those who stored their fat), (2) submuscular placement of a silicone gel implant, (3) vertical mastopexy, and/or (4) breast subunit reduction and/or skin tightening using VASER and HBT.

## 3. Results

**Figure 3:**
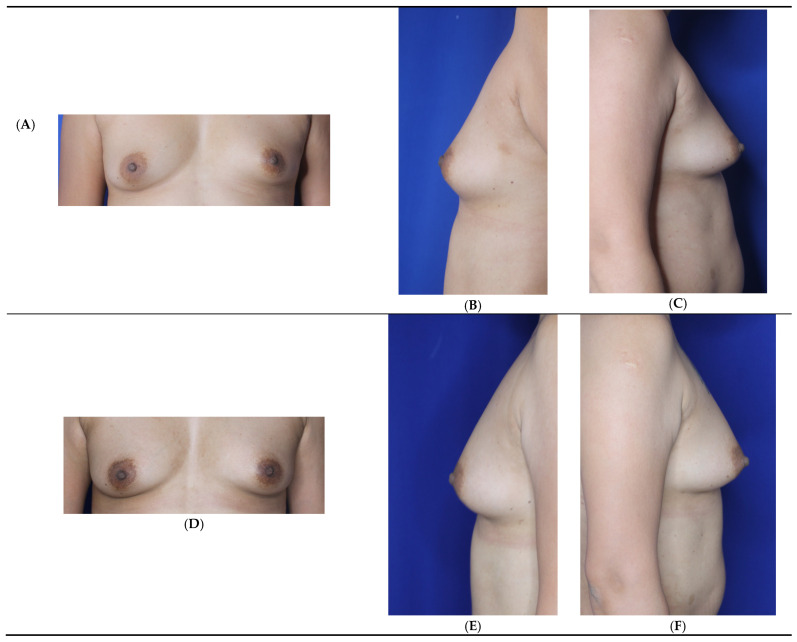
Primary breast augmentation by fat grafting in a 37-year-old woman G 2P2 (5′4″, 55.4 kg) who refused alloplastic implants, requesting fat grafting to the breasts. She had 1800 cc of fat removed by VASER liposuction from the abdomen, flanks, lower back, inner thigh, outer thigh, and axilla. She had simultaneous gluteal, hip, and right thigh crease fat transfer. She had 200 cc placed in each breast, all in the subcutaneous plane. She experienced no postoperative complications. Before (**A**–**C**). After (6 months) (**D**–**F**). (Courtesy of Troell, R).

**Figure 4:**
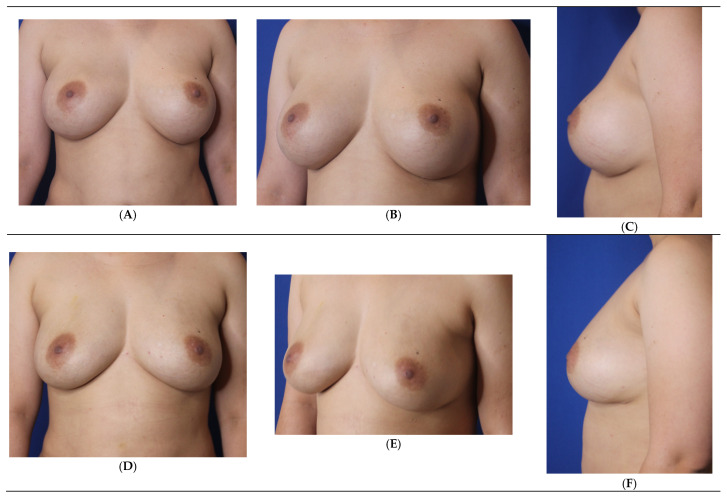
Secondary breast augmentation via simultaneous implant exchange with fat (SIEF). A 32-year-old G2P2 woman (5′3″, 71.8 Kg) requested removal of bilateral silicone gel high-profile breast implants (330 cc) placed in the dual plane. Simultaneously, she had a breast augmentation by autologous breast fat grafting using centrifuged filtered fat enriched with platelet-rich plasma and stem cells. She had 1100 cc of fat removed by VASER liposuction from the abdomen, flanks, lower back, and axilla. She had 240 cc injected into the left breast and 220 cc into the right breast, all placed in the subcutaneous plane. She had no postoperative complications. Before (**A**–**C**). After (**D**–**F**). (Courtesy of Troell, R).

**Figure 5:**
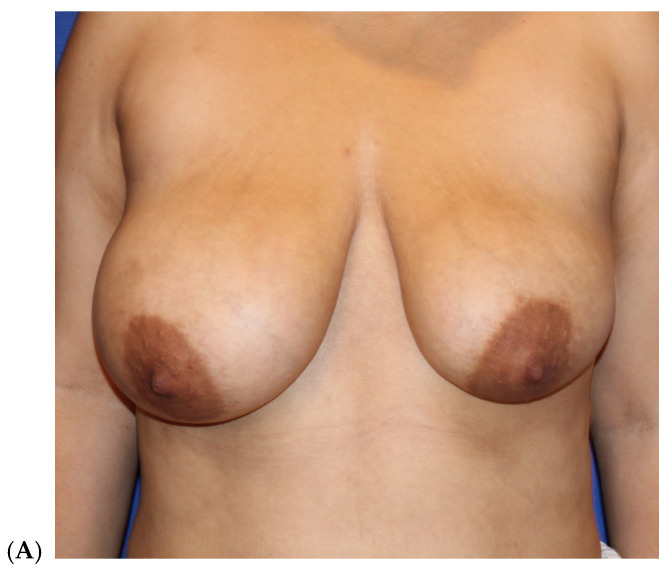

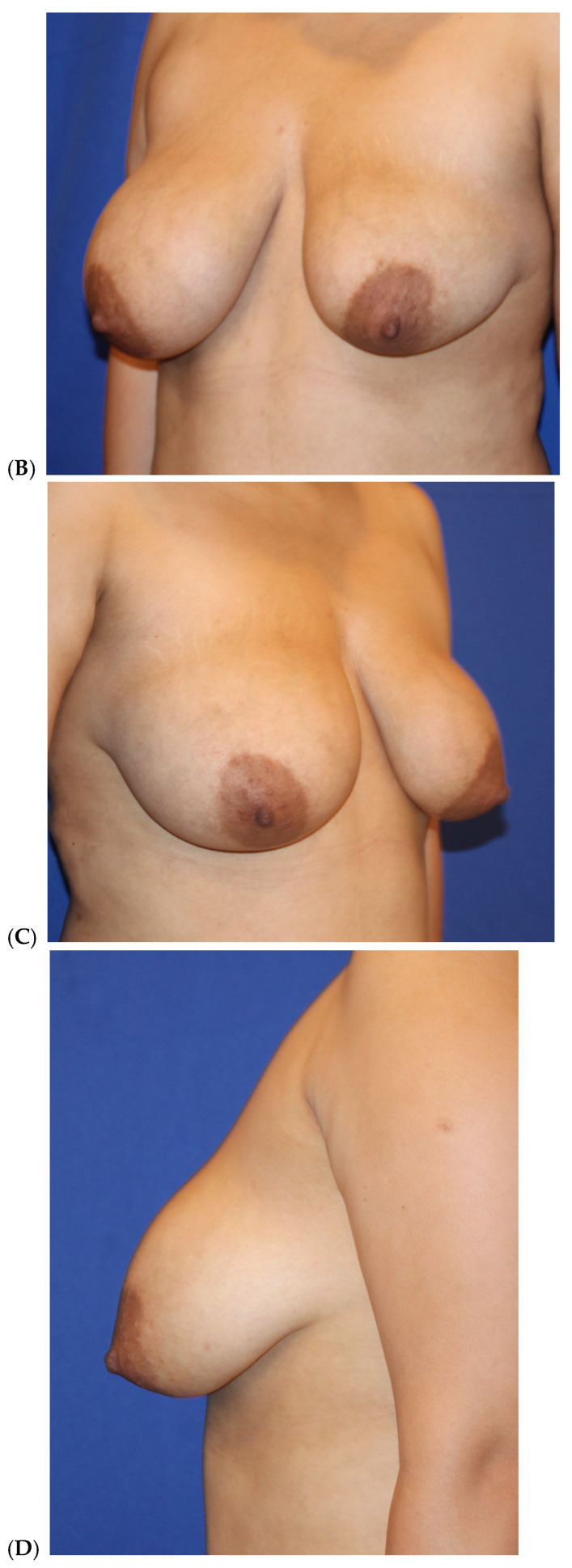

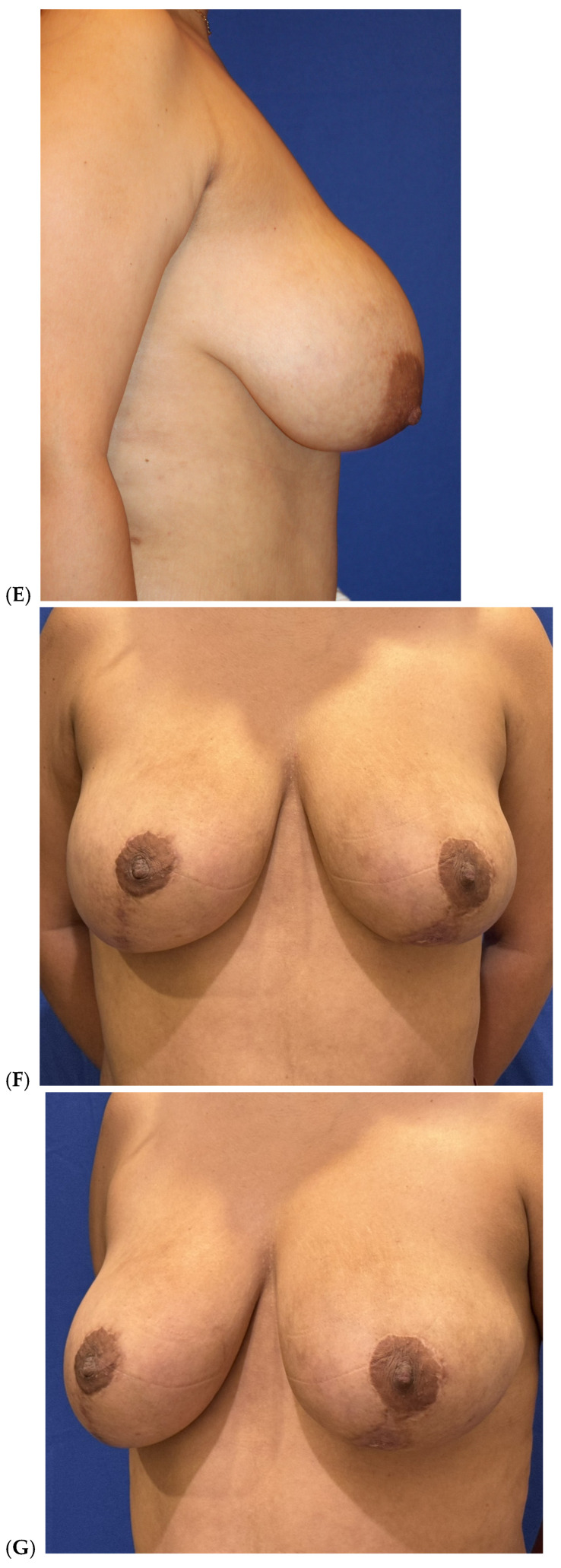

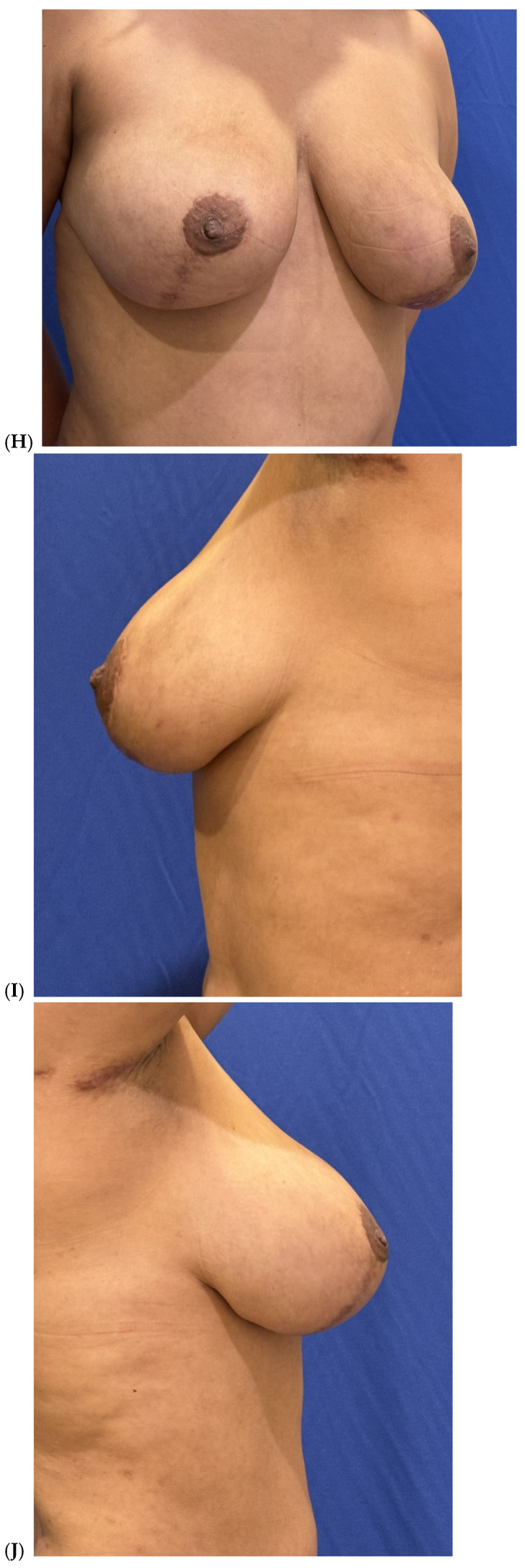
Secondary breast augmentation by fat grafting after mastopexy in a 33-year-old woman G2P2 (5′8″, 76 kg) who had undergone a vertical mastopexy along with right breast liposuction for grade III ptosis and significantly asymmetric breasts. She had a secondary breast augmentation two months thereafter using centrifuged filtered fat enriched with platelet-rich plasma and stem cells. She had a total of 300 cc of fat injected into the left breast and 100 cc into the right breast, all in the subcutaneous plane. Simultaneously, she had gluteal and hip fat grafting with high-definition VASER liposuction of abdomen, flanks, lower back, arms, and upper back. She was displeased with the left breast vertical limb mastopexy incision and underwent scar revision. One year after the original mammoplasty, a second session of fat grafting was added to the breast: 160 cc to the left breast and none to right breast. Before (**A**–**E**). After (16 months) (**F**–**J**). (Courtesy of Troell, R).

Patients electing breast fat transfer (n = 118) in this 15-year learned breast fat grafting clinical study between 2008 and 2025 were analyzed (Table 5) [6]. The most common indication for breast fat grafting was for primary breast augmentation (Figure 3) followed by fat grafting after implant removal (Figure 4, Table 6). The next most frequent indication is after a mammoplasty to optimize the end aesthetic breast appearance (Figure 5). The frequency of breast fat grafting cases ranged from 5–11 per year.

All patients (n = 118) had 360-degree VASER liposuction (abdomen, flanks, lower back), most (n = 62) had lateral chest/axilla liposuction, and some added (n = 17) Renuvion for optimal body contouring skin retraction.

The minimum amount of fat grafted was 80 cc per breast, with a maximum volume of 380 cc per breast. Fat was placed in the subcutaneous plane in all patients. In those who requested a larger breast size, had additional fat available to transfer, and did not have a contraindication for submuscular placement, between 40–180 cc of fat was placed below the pectoralis major muscle. The total mean volume per breast in the first 83 women was 190–195 cc of purified and enriched fat [6]. The final 35 women in this series had a total mean fat volume placed of 206 cc. About 10% of patients undergoing breast fat grafting also had hip and/or gluteal fat grafting, which limited the amount of fat available for breast transfer.

The observed complication rate for the breast fat grafting cohort was acceptable at 11.9% with no differences between the clinical indication groups and the majority related to the body liposuction, with a 7.6% (n = 9) incidence of seroma formation. Palpable fibrotic areas were second in frequency at 3.4% (n = 4), but with no instances of breast oil cyst formation (Table 7). Those with firm areas were successfully treated with triamcinolone injection to soften the firm areas.

A breast cancer survivor (74 y/o) had approximately 75% unilateral grafted fat absorption, suspected to be an atypical mycobacterial infection, although no growth in culture. She was treated with 6-week course of trimethoprim–sulfamethoxazole for suspected atypical mycobacteria with complete resolution of the inflammation and suspected infection.

### 3.1. Patient Satisfaction Assessment

Patient satisfaction analysis using the GAIS observed that 55.9% (66/118) were very satisfied, 41.5% (49/118) were satisfied, and 4.2% (5/118) were dissatisfied. The response of dissatisfaction was universally due to a smaller breast size than desired. The reason in four of the five dissatisfied patients was insufficient breast volume or breast asymmetry. Only one patient was dissatisfied secondarily due to a complication (atypical mycobacteria infection).

### 3.2. Revisions (7.6%, n = 9)

Those patients (n = 4) who opted for storage of their unused harvested fat at a cryobank elected an additional procedure to augment the size of their breasts. The other five patients requesting additional breast volume, harvested fat from areas not previously treated. We waited a three-month minimum before replantation. The mean fat administered in revision cases was 98 cc per breast with a maximum volume of 220 cc, all placed in the subcutaneous plane. Eight of the nine patients who had a revision were satisfied (88.9%) with their breast volume size.

One patient elected a silicone breast implant to achieve her desired size, while no patients required or elected a mastopexy. Some patients commented on the potential desire for a larger breast size (number not recorded), but either had insufficient fat to graft or did not desire breast implant surgery.

## 4. Discussion

Breast fat grafting is a valuable alternative to implant surgery and has numerous breast reconstruction clinical indications. However, to attain superior aesthetic results, minimize complications, and have a low revision rate requires using evidence-based medicine to optimize the harvesting, processing, enrichment, and administration of grafted fat. The fat grafting technique can be implemented anywhere in the body that fat grafting is contemplated, including the face, breast, buttock, hip, cellulite areas, hands, and calf.

The optimal breast fat grafting method in augmentation or reconstruction defined here has been garnered through evidence-based surgical experience. This optimal surgical technique employs third-generation ultrasound in pulsed mode at 60% energy with minimal duration of delivery [14,23,24], fat condensation and purification using centrifugation–filtration at 3000 rpms for 3 min with a 100 μ filter [14,28], enrichment with autologous adipose-derived stem cells and stromal vascular fraction support cells (one ml per 25 cc of processed fat) and platelet-rich plasma (one ml per 25 cc of processed fat) [6,14,35,36,37,39], fat administration within 1–2 h of harvesting [6,45], and injection in the subcutaneous plane in all patients and into the submuscular plane in those without contraindications with micro-aliquot quantities in a multi-plane injection technique [6,14,29,30,46,47].

This clinical experience did not use the BRAVA pre-expansion device because of the following reasons: (1) the literature did not reveal results superior to this experience implementing regenerative biologics (stem cells and growth factors), (2) the procedure would require a second surgery for all patients, (3) the expansion is time consuming and painful, and (4) the complication rate with BRAVA is more than with fat grafting alone. Superior long-term grafted fat volume was directly related to surgical technique best practices at each stage of the breast fat grafting process, highlighted by regenerative biologics added to purified fat.

In this experience, palpable fibrotic areas were noted in 3.4% (n = 4), but with no patients experiencing a breast oil cyst. Each small firm area was successfully treated by triamcinolone steroid injections to a soft texture. The reason for this low-level incidence was directly related to the premise of only administering purified enriched fat and limiting the amount of fat grafted into a specific extracellular matrix (ECM) area. The majority of oil cysts in the breast and buttocks are due to excess fat grafted to an area with insufficient ECM to propagate neovascularization.

Compacted purer fat grafts containing lower levels of oil, red blood cells, damaged adipocytes, and wetting solution fluid, avoiding the washing away of cellular growth factors, and enrichment with ASCs, SVF cells [35,36,39,44,45], and PRP [6,32] most likely contribute to improved graft take through superior neovascularization. Additionally, the centrifugation step, besides compacting fat and acquiring ASCs and SVF cells, removes cellular toxins, lipids, and both protease and lipase enzymes that may injure fragile adipocytes [28].

This fat purification and enrichment technique provides better predictability and consistency with 75–85% of augmented volume persisting long-term with less incidence of breast oil cysts (none in this study), fibrotic sites, and micro-calcifications [6,14]. Other surgeons have performed breast fat grafting with similar excellent outcomes and minimal complications, implementing nearly the same processing, enrichment, and administration technique [28,35,39]. Delay et al. published a similar clinical breast fat grafting experience using a similar technique (but without stem cell, SVF, or PRP enrichment). Their ten-year year cohort study yielded a high patient satisfaction rate with minimal observed minor complications [56].

Combining implants with fat grafting, referred to as “composite” fat grafting, is excellent for (1) improving soft tissue thickness to provide appropriate coverage of an implant, (2) camouflaging visible implant edges, (3) enhancing or improving the breast shape, and (4) balancing shape and size breast asymmetries.

Another clinical indication for reconstruction using stem cell-enriched fat grafting is for aesthetic augmentation after breast implant removal. A study by Ohashi et al. analyzed their technique and results with implant removal and simultaneous fat grafting. Our patients had a mammogram for cancer surveillance, while their patient group underwent diagnostic ultrasound to determine the presence of glandular micro-calcifications and implant characteristics including position, implant shell integrity, and depth [57].

They concluded that the key to a successful outcome was minimal tissue trauma during explantation, optimal fat processing, and micro-aliquot fat injection into multiple layers. Ohashi et al. had an even higher patient satisfaction rate than our study, most likely related to both more grafted fat (mean 240 cc per breast compared to our range of 190 to 206 cc) and the Japanese female population goal of a smaller breast size than women in the US [57].

Another concern of both cosmetic surgeons and breast cancer surgeons is the glandular oncologic concerns using stem cell-enriched fat grafting. Valente et al. showed in vitro studies that ASCs has a dual role in breast cancer, influencing proliferation, migration, and drug resistance through complex signaling pathways. In clinical patients, ASC-enriched fat grafting was safe with no increased cancer recurrence risk [58]. A meta-analysis on the oncologic safety of breast fat grafting by Tukiama et al. revealed no increased oncologic risk [59]. Furthermore, Kempa et al. followed 93 patients undergoing fat transfer reconstruction after breast cancer surgery. Their results showed one local recurrence (1.1%), 2 distant metastasis (2.2%), and 1 tumor-related death (1.1%) over a 12-year period. The authors concluded that breast fat grafting was a safe reconstructive option after breast cancer treatment [60]. Despite these clinical studies noting no increased cancer recurrence risk, there is always a concern of an increased oncologic risk when dealing with stem cells that promote cellular growth.

Besides the oncologic risk when employing autologous adipose-derived stem cells, physicians are concerned with the regulatory authority’s position. The FDA allows the use of autologous stem cell-enriched adipose tissue for fat grafting to the breast [13]. The US FDA published Regulatory Considerations for Human Cells, Tissues and Cellular and Tissue-Based Products: Minimal Manipulation and Homologous Use Definitions in 2020 (1271.10(a)(2) (21 CFR 1271)), which approves the use of homologous, minimally manipulated, and expanded fat for “systemic” use if the following requirements are fulfilled: use of an approved good tissue practice (GTP) laboratory, presence of a clean room, bacterial contamination testing, proper expansion methodology, clear client data identification, and use of a 170–200 μm blood filter for parenteral administration [13].

FDA regulations do not apply to products that fall outside the definition of HCT/P in 21 CFR 1271.3(d). Platelet-rich plasma (PRP) is not regulated, because it is a blood product, falling outside the FDA scope of this guidance. Recently, the State of Florida passed a bill to use non-FDA approved stem cell treatments for orthopedic, wound care, and pain management indications within the physician’s scope of practice [13,31].

Identification of mesenchymal stem cells that are autologous and derived from primary cell lines for expansion should follow the criteria established by the International Society for Cellular Therapy (ISCT). One of the few GTP stem cells banks in the United States is American Cell Technology (ACT, Sunrise, FL, USA), which performs regular validations to show that the expanded stem cells retain all their inherent cell surface markers and differentiation potential through flow cytometry analysis and tri-differentiation assays. The cell differentiation reveals CD 73 expression on 98%, CD 90 on 97%, and CD105 on 95% of stem cells and the absence of negative markers including CD34, C45, and CD19 showing less than 2% expression.

Additionally, ACT performs viability and cell counting along with sterility testing for aerobic, anaerobic, fungal, and mycoplasma cultures and endotoxin assays. This methodology maintains the highest standards of purity and identity, which is essential for efficacy and safety in practiced clinical applications. The initial 20 to 50 cc harvested fat sample is initially treated with collagenase and washed three times with a concentration of stem cells of 1–3 million ASCs per one cc of fat, which is expanded to 50 to 100 million ASCs in 3 to 7 days, depending on the donor patients’ demographic variables (ACT internal data). It is also of critical importance to maintain cell integrity and viability during the shipping process back to the physician for clinical use.

The complication rate of breast fat grafting is related to both patient variables as well as the fat grafting methodology. Related to the surgical purification and enrichment technique, no patients in our study formed a breast oil cyst, illustrating excess fat was not injected and that the processed and enriched grafted fat received sufficient vascularization. This illustrates the importance of an optimal fat grafting specimen containing live adipocytes and regenerative cells and growth factors absent oil, other blood cells, enzymes, and destructive cytokines. Also important are the administration of fat without excess injected volume using micro-aliquots and the multiple plane approach into a host with optimal characteristics including a history of no smoking or chronic steroid use, no or controlled diabetes mellitus, the absence of cardiovascular, collagen vascular, or autoimmune disease, younger age, and a recipient bed with lack of scarring and adequate extracellular space matrix thickness.

Preoperative risk factors are paramount to minimize complication rates. Nguyen et al. discovered that smoking was the only independent risk factor for infection and overall complications comparing breast augmentation with implants versus fat grafting [61]. The infection rate in the fat grafting group (n = 789) was 1.1% and 0.5% in the breast implant population (n = 18,544) [61]. Although not elucidated, besides increasing the risk of infection, smoking also inhibits neovascularization, which leads to fat necrosis, fibrotic areas, and microcalcifications.

Except for one patient who had suspected atypical mycobacteria observed over three months postoperatively, no patients had an infection. The keys to minimizing the infection risk are not operating on patients unless they stop smoking or vaping for a minimum of 2–3 weeks prior and 3–4 weeks after the procedure, perioperative antibiotics for one week, chlorhexidine skin preparation, a diligent intraoperative sterile technique, covering 2 mm breast incisions with band aids for one week postoperatively, and avoiding over-filling any area with grafted fat.

**Figure 6:**
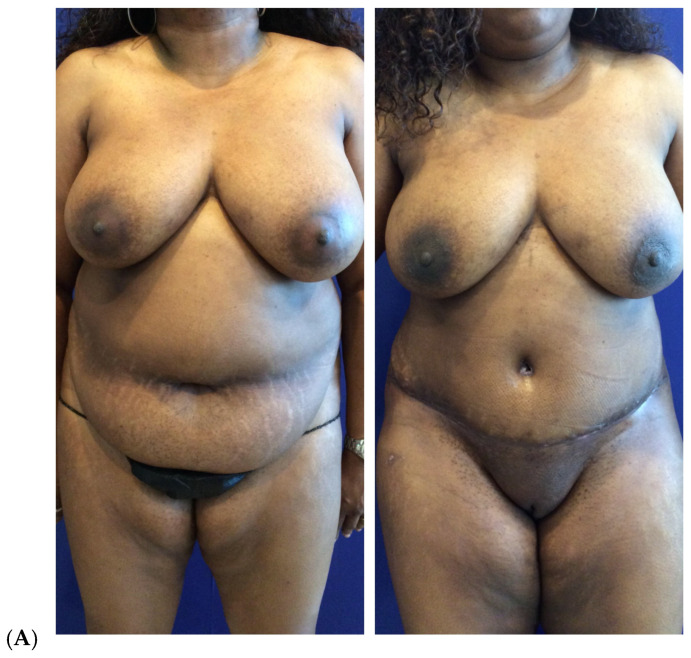

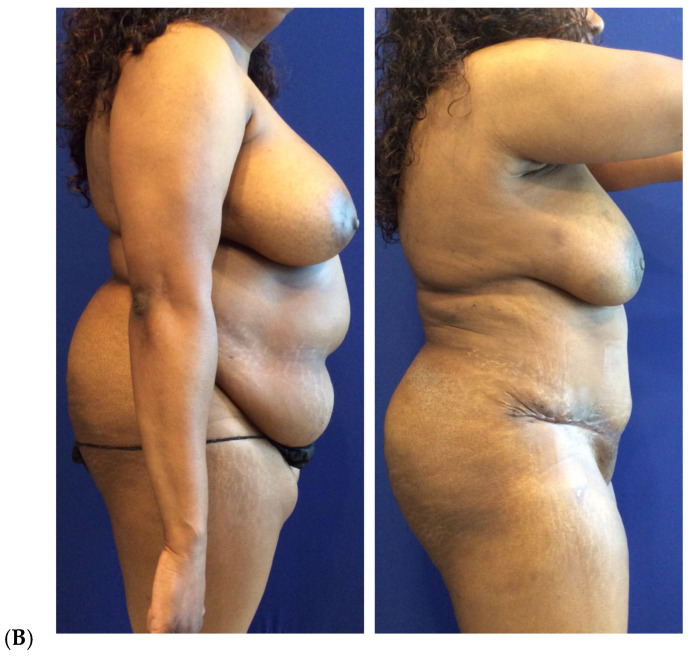
VASER liposuction breast reduction in a 48-year-old woman, Gravida 5, 5′5″, 192 pounds, who underwent lipoabdominoplasty with flank, lower back, middle back, and upper back liposuction and breast reduction using VASER liposuction. (**A**). Before and after front view (**B**). Before and after right-side view. (Courtesy of Troell, R).

Related to using technology (VASER ultrasound and HBT) to create both chest and breast skin tightening and breast reduction either to the overall breast or subunits, the author has used these successfully alone and in combination (Figure 6). Confirming the effectiveness of this approach, Sterodimas et al. implemented HBT as a minimally invasive mastopexy technique in a prospective study (n = 15). The results revealed no adverse effects with about a 1.0 cm breast lift, with all patients noting improved confidence and quality of life on the Breast-Q scale from more youthful-appearing breasts. Patients and the surgeon also noted an improved nipple position with less sagging, and the breasts had a higher appearance on the chest wall [62].

To provide simultaneous high-definition body contouring aesthetic outcomes, VASER ultrasound muscle sculpting not just harvests fat, but yields a superior athletic body appearance [22,26,63]. Ultrasound assists in separating adipocytes from their tissue matrix with minimal cell membrane lysis and has no effect on graft viability (when low energy levels are delivered) [24].

The studies of Schafer [23], Troell [14], and Fischer [24] have shown that decreasing ultrasound energy to 60% for a limited duration spares both adipocytes (87–92%) and stem cells (87–97%), with a significant volume of permanent fat grafting volume (75–85%). From previously analyzing cellular survival, we discovered that ultrasound-assisted harvesting combined with centrifugation–filtration processing yielded a mean fat viability of 91.5% with 7.9 × 10^5^ cells/mL compared to suction-assisted harvesting combined with centrifugation–filtration processing, which yielded a mean fat viability of 92.1% with 5.7 × 10^5^ cells/mL [14].

VASER creates tightening by myofibroblast stimulation and the healing response in contrast to heat alone [26]. Helium-based plasma technology uses heat-based collagen fiber contraction [51,52,53]. Troell and Javaheri’s data show that combined VASER with optimal energy delivery of Renuvion, performed using 6 passes with the 60–80% RF power settings, moving the piece at no more than 3 cm/second, can be performed safely, achieving superior skin contraction [62]. There were 561 anatomic liposuction areas in 160 patients, with an average of 3.5 sites per patient. The majority of patients (90.6%) had 360° (abdomen, flanks, and lower back) liposuction. The overall patient satisfaction rate was 95% (152 of 160 patients) [63].

Maximizing skin tightening is achieved by manual suction liposuction creating 8–10% skin retraction [64], ultrasound energy delivery with an estimated 20% skin tightening [26,63], and helium-based plasma technology with an estimated 20 to 30% more retraction [51,52,53,54,63].

### Study Limitations

Study limitations including not being prospective, randomized, or blinded without a control group; however, the advantage of this retrospective study is the application of consecutive patients without bias or exception. As with most clinical patient analyses, the numbers of patients could have been larger for more statistical power. The patient satisfaction rate or patient reported outcome measure was the main outcome metric used in this clinical study, implementing the standardized and validated Global Aesthetic Improvement Scale (GAIS) assessment, since pleasing the individual patient to their aesthetic desires was our clinical goal.

The advantage of a single-surgeon study was that the surgical technique and protocol was nearly identical in each patient. Multi-center study advantages are that they provide external validity, yet the variations in technique limit a direct outcome comparison. The addition of similar body contouring clinical studies regarding their similar outcomes referenced in the discussion section assists in validating the presented results.

Subjective aesthetic outcomes were presented without blinded assessments; however, patient, surgeon, and surgical team individual observations were congruent. Objective results were presented using three-dimensional imaging (diagnostic ultrasound) for volume assessment and objective adipocyte and stem cell quantitative laboratory analysis from two good manufacturing practices (GMP) laboratories were carried out on a sub-population of patients, although this was not implemented in all patients.

Clinical studies evaluating fat grafting outcomes can increase the academic value by implementing validated patient and surgeon satisfaction assessments, considering the addition of three-dimensional imaging techniques, such as MRI [14,65,66], and using blinded evaluators to assist in objectively analyzing cosmetic results.

## 5. Conclusions

Primary breast augmentation and other breast reconstruction indications using autologous fat grafting are now commonplace. Evidence-based medicine has defined the optimal surgical techniques and adjuvant technologies yielding superior adipocyte survival in breast fat grafting regarding harvesting, processing, enrichment, storage, and administration. Implementing minimally traumatic harvesting techniques, fat purification and condensation using centrifugation–filtration at 3000 rpms for 3 min with a 100 μ filter, and autologous stem cells (ASCs), stromal vascular fraction (SVF), and platelet-rich plasma (PRP) growth factor enrichment provides consistent, superior breast enhancement aesthetic results. Additionally, simultaneous body contouring outcomes using ultrasound-assisted (VASER) high-definition liposuction, helium-based plasma technology (Renuvion), and a plethora of available body silastic implants alone or combined with fat grafting provide unsurpassed body contouring aesthetic outcomes.

## Figures and Tables

**Table 1:** Breast fat grafting clinical indications.

Primary breast augmentation Simultaneous implant exchange with fat (SIEF) Improve shape and/or volume post-mastopexy Improve shape or volume post-reduction mammoplasty Improve shape and/or volume post-implant placement Complete breast augmentation after mastectomy Treatment of implant-related capsular contracture Fill isolated volume breast defects Asymmetric breasts (natural or iatrogenic) Tuberous breasts Repair chest wall defects

**Table 2:** Medical issues lowering breast fat graft survival.

HIV disease Collagen vascular disease Autoimmune diseases, such as rheumatoid arthritis Previous and (more impactfully) current smoking history Diabetes mellitus (especially if hemoglobinA1C > 7.0) Underlying cardiovascular disease Chronic steroid use Increased patient age (>60 years of age) Previous breast radiation therapy Breast implant capsule presence

**Table 3:** Harvesting variables effecting adipocyte survival.

Liposuction technique: Suction at ½ atm (<−18 in Hg) Use ≥ 3.0 mm sized harvesting cannulas Vented harvesting cannulas preferred Closed fat container (avoid fat exposure to air) . Ultrasound (VASER) energy delivery 60%—pulsed mode for minimal time duration. . Donor site does not affect survival, but inner thigh has observed less degree fibrous tissue.

VASER (vibration amplification of sound energy at resonance). Third-generation ultrasound machine for liposuction surgery.

**Table 4:** Processing, enrichment and administration affecting outcomes.

Proper identification of recipient area for fat grafting Pre-tunneling of recipient area soft tissues Minimal washing of fat (removes less growth factors) Centrifugation–filtration extraction of maximum fluid, blood, and oil (3000 rpms for 3 min, 100 μ filter Enrichment with platelet-rich plasma (PRP) Enrichment with autologous stem cells and SVF Micro-aliquot injection Multiple tissue plane depth placement Administration within two hours of harvesting Administration cannula > 3.0 mm diameter best Less volume grafted, greater adipocyte survival rate Subcutaneously preferred, then submuscular placement

SVF—stromal vascular fraction (support cells for stem cells).

**Table 5:** Patient demographics (N = 118).

Sex: All women Age: 22–75 years Height: 5′1″–5′10″ Weight: 46–106 kg BMI: 19.4–33.3 Fat volume (mean): 192.3 cc (first 83 patients) Fat volume (mean): 206.2 cc (last 35 patients)

(30 cc centrifugation–filtration volume = 50 cc gravity-separated fat). 206.2 cc × 1.67 (60% less cc volume with purified, compacted fat) equals 343.7 cc of gravity-separated fat volume.

**Table 6:** Breast fat grafting 15-year clinical patients (N = 118).

Procedure Indications	Number of Cases
Primary aesthetic breast augmentation	67
Fat grafting after implant removal (staged)	16
Simultaneous implant exchange with fat (SIEF)	15
During or after breast lift/reduction	16
Breast cancer reconstruction	4

**Table 7:** Breast fat transfer complications (N = 14).

Complication	Number
Seroma ^τ^	6
Hematoma	0
Palpable fibrotic areas ^Ω^	4
Breast oil cysts	0
Bacterial infection ^¶^	1
Nipple–areolar vascular compromise ^€^	1
Scar revision of mammoplasty incision	2

^τ^—Seroma noted at body liposuction/fat donor sites. ^Ω^—All palpable fibrotic areas resolved after 1–2 injections of either. Triamcinolone alone or combined with 5-flurouracil. ^¶^—Suspected atypical mycobacterial infection persistent soft tissue erythema. No growth in culture, treated with 6-week duration of sulfamethoxazole/trimethoprim with complete resolution. Asymmetric grafted fat absorption as a sequela to infection. ^€^—Breast fat grafting performed ten days before vertical mastopexy with a 9 cm pedicle length. Venous compression most likely the cause of compromise and not due to fat transfer.

## Data Availability

Patient data were compiled from patient charts. The data were placed into tables for analysis.
